# EDIL3 influenced the αvβ3‐FAK/MEK/ERK axis of endothelial cells in psoriasis

**DOI:** 10.1111/jcmm.17544

**Published:** 2022-09-06

**Authors:** Xuping Niu, Qixin Han, Xinhua Li, Juan Li, Yanmin Liu, Yan Li, Yan Wu, Kaiming Zhang

**Affiliations:** ^1^ Shanxi Key Laboratory of Stem Cells for Immunological Dermatosis, Institute of Dermatology Taiyuan Central Hospital of Shanxi Medical University Taiyuan Shanxi China; ^2^ No. 1 English Department, School of Fundamental Sciences China Medical University Shenyang China; ^3^ Department of Dermatology Key Laboratory of Immunodermatology, Ministry of Education and NHC National joint Engineering Research Center for Theranostics of Immunological Skin Diseases The First Hospital of China Medical University Shenyang China

**Keywords:** dermal mesenchymal stem cells, EDIL3, endothelial cells, integrin, psoriasis

## Abstract

One of the earliest events in the development of psoriatic lesion is a vascular network expansion. The abnormal vascular network is associated with increased endothelial cells (ECs) survival, proliferation, adhesion, migration, angiogenesis and permeability in psoriatic lesion. Our previous study demonstrated that epidermal growth factor‐like repeats and discoidin I‐like domains 3 (EDIL3) derived from psoriatic dermal mesenchymal stem cells (DMSCs) promoted cell–cell adhesion, migration and angiogenesis of ECs, but the molecular mechanism of upstream or downstream has not been explored. So, this study aimed to explore the association between EDIL3 derived from DMSCs (DMSCs‐derived EDIL3) and psoriasis‐associated angiogenesis. We injected recombinant EDIL3 protein to mouse model of psoriasis to confirm the roles of EDIL3 in psoriasis. Besides, we employed both short‐interference RNA (si‐RNA) and lentiviral vectors to explore the molecular mechanism of EDIL3 promoting angiogenesis in psoriasis. In vivo, this research found that after injected recombination EDIL3 protein, the epidermis thickness and microvessel density were both elevated. EDIL3 accelerated the process of psoriasis in the IMQ‐induced psoriasis‐like mouse model. Additionally, we confirmed that in vitro DMSCs‐derived EDIL3 is involved in the tube formation of ECs via αvβ3‐FAK/MEK/ERK signal pathway. This suggested that DMSCs‐derived EDIL3 and αvβ3‐FAK/MEK/ERK signal pathway in ECs play an important role in the pathogenesis of psoriasis. And the modification of DMSCs, EDIL3 and αvβ3‐FAK/MEK/ERK signal pathway will provide a valuable therapeutic target to control the angiogenesis in psoriasis.

## 1 INTRODUCTION

Psoriasis is a common skin disorder that is associated with both a physical and psychological burden.^1^ The worldwide prevalence is about 2%.^2^ Histopathology revealed keratinocyte hyperproliferation, infiltration of inflammatory cells, dilatation and tortuous of dermal papillary blood.^3^ One of the earliest events in the development of psoriatic lesion is a vascular network expansion, which occurs before epidermal changes and persists after clearance of the clinical lesions.^4^ The dilatation and tortuous of vascular network is associated with increased endothelial cells (ECs) proliferation, adhesion, migration, angiogenesis and permeability in psoriatic lesions.^5^, ^6^, ^7^, ^8^ Recently, researchers found that the dermal mesenchymal stem cells (DMSCs) in psoriatic lesions produced more angiogenic and pro‐inflammatory mediators and involved in course of psoriasis.^9^, ^10^, ^11^ Our previous study demonstrated that psoriatic DMSCs promoted cell–cell adhesion, migration and angiogenesis of ECs, but the molecular mechanism of upstream or downstream has not been explored.^12^, ^13^

Epidermal growth factor‐like repeats and discoidin I‐like domains 3 (EDIL3) is an extracellular matrix protein, also named as developmental endothelial locus‐1 (Del‐1), composed of two discoidin I‐like domains and three EGF‐like repeats, the second of which contains an Arg‐Gly‐Asp (RGD) motif.^14^ The RGD motif can bind with integrin, then effect ECs functions including adhesion, migration and angiogenesis.^7^, ^9^, ^14^ During angiogenesis in early embryogenesis,^7^ tumour^15^ and ischemic tissue,^16^ EDIL3 is an important molecular for mediating ECs functions. Our prior study demonstrated the mRNA and protein expression of EDIL3 in DMSCs was significantly upregulated in psoriasis.^9^ In vitro, EDIL3 derived from DMSCs (DMSCs‐derived EDIL3) in psoriasis promotes angiogenesis of ECs.^13^

Based on previous research, we explored the mechanism of DMSCs promoting angiogenesis in psoriasis. First, we injected recombinant EDIL3 protein to mouse model of psoriasis to confirm the roles of EDIL3 in psoriasis. Besides, we employed both short‐interference RNA (si‐RNA) and lentiviral vectors to explore the molecular mechanism.

## 2 MATERIALS AND METHODS

### 2.1 Mice and treatment

All procedures involving in mice experiments were approved by the Animal Care and Use Committee of the Shanxi Medical University in conformity with National Institutes of Health guidelines. BALB/c mice at 6‐8‐weeks of age were purchased from Changsheng Biological Corporation. Mice were housed in standard sterile mouse cages. Mice were depilated on the back skin before the treatment using depilatory cream (Veet, Shanghai, China) and randomly assigned to different experimental groups in a blind manner. Grouping and processing are as follows: (1) mice were treated with 62.5 mg of Vaseline cream on the shaved back for 7 consecutive days (Control, Mingxin, Sichuan, China); (2) mice received a daily topical 62.5 mg dose of 5% Imiquimod cream on the back for 7 consecutive days (IMQ; Mingxing); (3): mice received a daily topical 62.5 mg dose of 5% IMQ cream and subcutaneous injection 50‐μl NS solution on the back for 7 consecutive days (IMQ+ 0.9% NS); (4) mice received a daily topical 62.5 mg dose of 5% IMQ cream and subcutaneous injection of 50‐ul EDIL3 protein on the back for 7 consecutive days (IMQ+ 20 ng/μl EDIL3). Recombinant EDIL3 protein was obtained from R&D Systems. Disease severity of the back lesions was evaluated daily with a semi‐quantitative scoring system including scaling and erythema. To evaluate microvessel densities during modelling, at on day 4, half of every group mice were sacrificed by cervical dislocation and skin samples were collected within 2 h for additional experiments. At the end of the experiment on day 7, remainder mice were sacrificed and skin samples were collected. Skin biopsies were processed for paraffin sections, which were then stained with haematoxylin and eosin (H and E). Moreover, skin biopsies were embedded in tissue‐tek OCT compound (Thermo Scientific) and stored at −80°C.

### 2.2 Immunofluorescent and H and E staining

For measuring epidermal thickness, paraffin sections were stained with H and E. and images were taken by inverted microscopy (Olympus). Epidermal thickness was measured under microscope.

The immunofluorescent (IF) staining of mice skin biopsies for CD31 was performed to assess the microvessel densities. The 5‐μm thick tissues were sectioned from frozen tissue. To inhibit non‐specific antigen–antibody reactions, tissues were blocked with goat serum (Boster Biological Technology) for 30 min and washed for 3 times with PBS buffer. Primary rabbit anti‐CD31 (Abcam) was added and incubated at 4°C overnight. The next day, recovery temperature at 37°C, slides were washed in PBS for 3 times and incubated with secondary antibody (Goat anti‐rabbit IgG; Zhongshanjinqiao) for 2 h at room temperature. Finally, slices were washed for three times with PBS, incubated with 4′, 6‐diamidino‐2‐phenylindole (DAPI; Solarbio) for 10 min at room temperature. Sections were then imaged using LSCM (Olympus FV1200MPE).

### 2.3 Samples collection

10HEK293 cells were kindly donated by Dr. Ruixia Hou (Department of Dermatology, Taiyuan Central Hospital of Shanxi Medical University). DMSCs from five patients with psoriasis and five healthy volunteers were isolated from skin tissues and the isolated and identified methods have been described in previous work.^17^ The patients with psoriasis had been diagnosed both clinically and pathologically and received neither pharmaceutical nor physical therapy within least 12 weeks. Characteristics of psoriatic patients are shown in Table S1. The normal skin samples were collected from healthy volunteers undergone routine plastic surgery without cutaneous or inflammatory‐mediated diseases. HEK293 cells and DMSCs were incubated in Dulbecco's modified Eagle's media: Nutrient Mixture F‐12 (DME/F12; Hyclone) supplemented with 10% foetal bovine serum (FBS; Hyclone) and 1% penicillin/streptomycin (Solarbio) at 37°C in a humidified atmosphere containing 5% CO_2_. Human umbilical cord tissues from five caesarean sections were harvested for isolating primary human umbilical vein endothelial cells (HUVECs). This study was approved by the Ethics Board of Taiyuan Central Hospital in Shanxi Medical University (No. 2018010). Written informed consents were obtained from each volunteer prior to the study. The present experiments were performed in accordance with the Helsinki Declaration.

### 2.4 Cells isolation and identification

DMSCs were isolated and cultured as described previously.^17^ Briefly, skin tissue was cut into pieces and subcutaneous fat was removed. The dermis was separated from the epidermis by incubating in 0.25% dispase (Hyclone). Then, dermal pieces were chopped further and filtered. The suspension containing cells was collected. Finally, after centrifugation, cells pellets were seeded into a culture dish and cultured in DME/F12 supplemented with 10% FBS and 1% antibiotics. The non‐adherent cells were removed after 48–72 hours. The medium was refreshed every 5 days and the colonies of DMSCs appeared at 7 to 10 days. The phenotype of DMSCs was identified by flow cytometry at passage 3 using antibodies against human CD105, CD29, CD44, CD73, CD90, CD45, CD34 and CD14 (Becton Dickinson and Company). To further confirm the identification of cells, DMSCs at passage 3 were induced to differentiate into osteoblasts, adipocytes and chondrocytes according to our previously published study.^9^

HUVECs were isolated using a standard trypsin enzyme digesting technique. Human umbilical cords were harvested and immediately placed into DME/F12 (Hyclone) supplemented with 10% FBS (Hyclone) and 1% penicillin/streptomycin (Solarbio), stored at 4°C and used within 24 h. Firstly, to remove blood cells from the umbilical vein, we flushed the vein with warmed phosphate buffer solution (PBS; Solarbio) until the effluent buffer was transparent or slightly pink. Then, 0.25% trypsin solution was injected into the vein via syringe, and when the solution overflowed from the other end of the umbilical vein with a vessel clamp. Then continued to inject the trypsin solution and tightly clamped it with another vessel clamp until trypsin solution filled with the vein. Finally, the umbilical cord was incubated at 37°C. After 20 min, the clamp was opened and the cell suspension was collected. The vein was flushed twice with PBS to collect as many cells as possible. To harvest cells pellets, the cell suspension was centrifuged at 800 *g* for 5 min. The cells were resuspended and incubated in T25‐cell culture flask with 5 ml endothelial basal medium (EBM; Lonza) supplemented with EGM‐2 (Lonza) at 37°C in a humidified atmosphere containing 5% CO_2_. At 48–72 h, we observed the adherent cell islets under phase‐contrast microscopy, then, we removed non‐adherent cells by changing the culture medium. In passage 3–5, HUVECs were used to conduct subsequent experiments.

The purity of HUVECs was examined by flow cytometry. The surface marker platelet endothelial cell adhesion molecule‐1 (PECMA‐1; CD31) of ECs was detected. Cells were incubated with fluorescein isothiocyanate (FITC)‐conjugated mouse anti‐human CD31 (Abcam). The expression of CD31 on cells surface was analysed by flow cytometry (Beckman Coulter).

### 2.5 HUVECs co‐culture with DMSCs

Transwell chamber with 0.4 μm pore filters was selected to create an indirect interaction microenvironment for co‐culture. HUVECs were co‐cultured with DMSCs in a 12‐well transwell plate for 48 h at 37°C in 5% CO_2_. DMSCs (8 × 10^4^ cells/well) were seeded into the upper chambers in the transwell plates followed by 500 μl medium (10% v/v FBS in DME/F12). Then HUVECs were seeded into the lower chambers at 1:1 ratio (DMSCs: HUVECs) followed by 1000 μl EBM medium supplemented with EGM‐2. Before HUVECs adhesion, DMSC‐seeded chambers were moved into new 12‐well plates for 6 h to exclude the influence that HUVECs adhered to the well plate. After co‐cultured, HUVECs were harvested for real‐time quantitative polymerase chain reaction (RT‐qPCR), western blot and HUVECs function analysis.

We divided HUVECs into five groups according to DMSCs co‐cultured with them. The details of groups were as follows: Control group: HUVECs were cultured in the lower chamber alone. C‐DMSCs group: DMSCs from healthy volunteers were co‐culture with HUVECs. C‐DMSCs^EDIL3‐high^ group: DMSCs from healthy volunteers with overexpressed EDIL3 co‐cultured with HUVECs. P‐DMSCs group: DMSCs from psoriasis co‐cultured with HUVECs. P‐DMSCs^EDIL3‐low^ group: DMSCs from psoriasis with low‐expressed EDIL3 co‐cultured with HUVECs.

### 2.6 Short‐interference RNA transfection to P‐DMSCs

For silencing EDIL3 of P‐DMSCs, EDIL3 si‐RNA (GenePharma) was transfected using HiperFect transfection reagent (QIAGEN) following the manufacturer's instructions. P‐DMSCs were seeded into a 6‐well plate until reached about 50% confluence and washed with DMEM/F12 for 3 times prior to transfection. Serum‐free DMEM/F12 was mixed with 8 μl of HiperFect transfection reagent and 4 μg of si‐RNA or negative control si‐RNA (NC) and incubated for 20 minutes at 37°C. Then the mixture was added into 6‐well plate containing of P‐DMSCs and cultured at 37 °C for 24 h. Media containing si‐RNA were removed and replaced by DMEM/F12 supplied with 10% FBS after transfected for 24 h. After 24 h, the silence efficacy was evaluated by Laser Scanning Confocal Microscopy (LSCM, Olympus FV1200MPE) and the photographs were gathered. At 48, 72 and 96 h, respectively, cells were harvested for western blot analysis to detect the expression of EDIL3. EDIL3 si‐RNA sequences are as follows: forward 5’‐GGUGAUAUUUGUGAUCCCATT‐3′ and reverse 3’‐UGGGAUCACAAAUAUCACCTT‐5′ and negative control si‐RNA: forward 5’‐UUCUCCGAACGUGUCACGUTT‐3′ and reverse 3’‐ACGUGACACGUUCGGAGAATT‐5′.

### 2.7 EDIL3 lentiviral vectors preparation and transduction to DMSCs

Recombinant EDIL3 lentiviral vectors were assembled by an EDIL3 expressed plasmid with green fluorescent protein reporter (pLenti‐CMV‐EDIL3‐Flag‐GFP‐Puro) and two lentiviral packaging plasmids: psPAX2 and pMD2.G (PPL). EDIL3 expressed plasmid and packaging plasmids transfected HEK293 cells by Lipofectamine 2000 (Signagen). After transfection for 48 h, fluorescence intensity of HEK293 cells was used to evaluate transfection efficiency. And cell supernatant containing recombinant EDIL3 lentiviral vectors were collected and purified using BW‐V2001 lentivirus concentration reagent (Biomiga) according to the manufacturer's instructions.

C‐DMSCs were cultured in T25‐culture flask with 5 ml DME/F12 containing 10% FBS. C‐DMSCs grown to 50% confluence were washed for three times by PBS and treated with recombinant EDIL3 lentiviral vectors. Quantification of cell fluorescence was performed using LSCM after 48 h. Infected cells were harvested for western blot analysis for detecting the expression of EDIL3.

### 2.8 Real‐time quantitative polymerase chain reaction (RT‐qPCR)

Transfected P‐DMSCs were harvested and mRNA was extracted using Trizol reagent (Invitrogen) according to the manufacturer's protocol. Then complementary DNA (cDNA) was reversely transcribed with mRNA and PrimeScript™ RT Master Mix kit (Takara). RT‐qPCR was performed in a gradient thermal cycler (Bio‐Rad) using primers and TB Green™ Premix Ex Taq (TaKaRa). The β‐actin was used as an internal control. The EDIL3 specific primers: forward 5’‐AGCATACCGAGGGGATACATT‐3′ and reverse 3′‐ CAAGGCTCAACTTCGCATTCA‐5′. The β‐actin primers: forward 5’‐CTACAATGAGCTGCGTGTGGC‐3′ and reverse 3’‐CAGGTCCAGACGCAGGATGGC‐5′. The expression level of the target gene was calculated according to the formulas: Target gene/ control = 2^–ΔΔCt^, where ΔΔCt = (Ct(target gene)—Ct(reference gene)) treat group—(Ct(target gene)—Ct(reference gene)) control group.

### 2.9 Western blot analysis

Cells were lysed in RIPA buffer (Beyotime) containing protease inhibitor PMSF (Solarbio) at ratio (100:1 = RIPA: PMSF) and protein phosphatase inhibitor (Solarbio), then clarified by centrifugation at 13,000 *g* for 10 min at 4°C. Protein concentration was quantified using BCA Protein Assay Kit (Solarbio, Beijing, China). Western blot was performed by automatic protein analyser (Wes&Jess) according to the manufacturer's instruction. All protein levels were normalized to β‐actin. The blots were reacted with rabbit anti‐human against β‐actin (CST), phospho‐FAK, FAK, phospho‐MEK1, MEK1, phospho‐ERK1/2, ERK1/2, β3, αv, α5, β1 (all from Abcam).

### 2.10 Tube formation assay

In vitro, the tube formation of ECs was assessed using Matrigel matrix (mimics the natural basement membrane matrix of ECs). All procedures were performed on ice under sterile condition. The 96‐well plate and micropipette tip were pre‐cooled. Then, 40 μl/well Matrigel was coated in 96‐well plate and placed at 37°C in a CO_2_ incubator for 3 h. After co‐culture, HUVECs were trypsinized and seeded at a density of 2 × 10^4^ cells/well in 100ul EBM medium containing EGM‐2. After 6 h, at peak of tube formation, the images of tube‐like structures were captured using an inverted microscope (Olympus). The mesh numbers of tube structures were measured using ImageJ software and the results were expressed as the mean ± SEM.

### 2.11 Statistical analysis

Statistical analyses were performed using 17.0 SPSS software (Chicago, USA). Differences among groups were evaluated using repeated measures analysis of variance (anova). A two‐tailed t‐test was used when two groups were compared for statistical significance. All data were expressed as mean ± SEM. p < 0.05 considered to be statistically significant (*p < 0.05, **p < 0.01, ***p < 0.001).

## 3 RESULTS

### 3.1 EDIL3 accelerated the process of psoriasis in vivo

**FIGURE 1:**
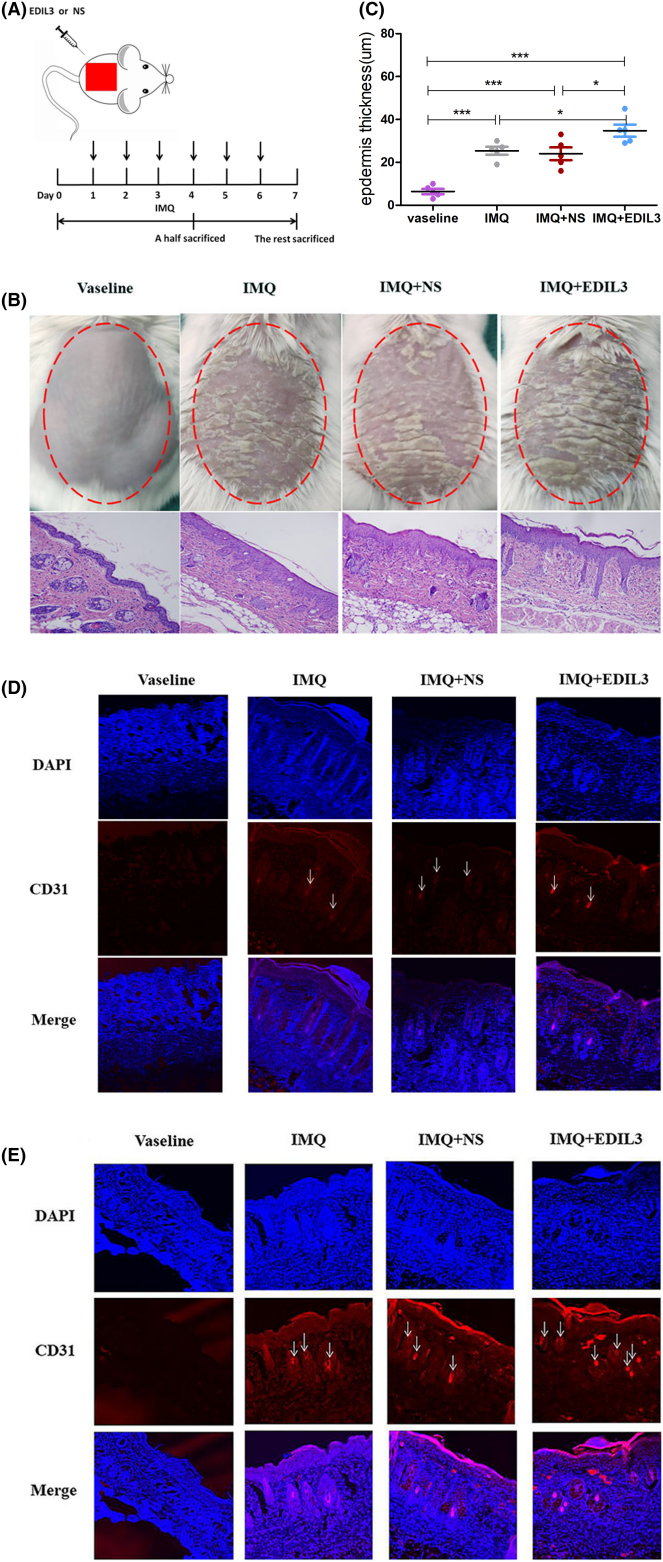
EDIL3 promoted dermal microvascular formation of psoriasis in vivo. (A) Schematic diagram for intradermal administration of EDIL3 or 0.9% normal saline (NS) on day 1–4 or day 1–6 during the application of IMQ in BALB/c mice. A half of mice in each group were sacrificed on days 4 and 7 to conduct experiments. (B) The clinical manifestations and H and E staining of the mice back skin treated for 6 consecutive days with Vaseline cream or IMQ and injected with EDIL3 or NS. (C) Epidermis thickness of the back skin was measured on the seventh day under a microscope. (D) The frozen sections of mice back skin were analysed using immunofluorescence staining for CD31 (red, ECs surface marker) and DAPI (blue, cell nucleus) on day 4. (E) The frozen sections of mice back skin were analysed using immunofluorescence staining for CD31 and DAPI on day 7. The arrows pointed to the microvessels (magnification 10×)

To further confirm the roles of EDIL3 in psoriasis. We intra‐dermally injected recombinant EDIL3 protein or 0.9% sodium chloride solution (NS) once per day for 3 and 6 consecutive days in the IMQ‐induced psoriasis‐like mouse model, and the mice were sacrificed at day 4 and 7 (Figure 1A). After administrating consecutively IMQ cream or Vaseline on the shaved mouse back skin for 7 days, we assessed whether IMQ application induced skin inflammation and hyperproliferation of epidermis accompanied by characteristic structural features of psoriasis and the clinical and pathological phenotypes of mice model were shown in Figure 1B. Mice treated daily with IMQ cream showed the same as psoriasis‐like lesion. Besides, we applied a semi‐quantitative scoring system from 0 to 4 based on their external physical appearance: 0, none; 1, slight; 2, moderate; 3, marked; 4, very marked; to accurately evaluate the clinical phenotype of mice model. The erythema and scaling scores of individual mice in every group were shown in Figure S1. The erythema and scaling scores of mice in groups had no statistical difference. But, histological analysis showed that epidermal thickness was markedly increased in IMQ‐treated mice compared with Vaseline‐treated (all *p* < 0.001) (Figure 1C). And interesting, IMQ + EDIL3‐induced psoriasis‐like lesion in mouse model resulted in higher epidermis thickness compared with the IMQ + NS‐treated (*p* < 0.05) (Figure 1C, Table S2). These results demonstrated that EDIL3 accelerated the pathological progress of IMQ‐induced psoriasis mice.

Researches showed that EDIL3 promoted tumours growth through promoted angiogenesis.^15^ To explore whether EDIL3 accelerated the pathological progress of psoriasis is also through modulated angiogenesis, we conducted IF analysis. We used double IF with DAPI and CD31 to detect the microvascular density via two‐photon confocal laser microscopy. We confirmed an upregulation of blood vessel density in psoriatic mice skin at day 4 and 7 (Figure 1D,F). And the results indicated that EDIL3 significantly promoted the growth of microvasculature. In addition, compared to that at day 4, EDIL3‐injected skins exhibited more increased blood vessel density at day 7, and EDIL3 caused a time‐dependent increase of angiogenesis. Together, these data suggested that the upregulation of EDIL3 can promote microvascular formation in development of psoriasis.

### 3.2 Identification and culture of DMSCs and HUVECs

**FIGURE 2:**
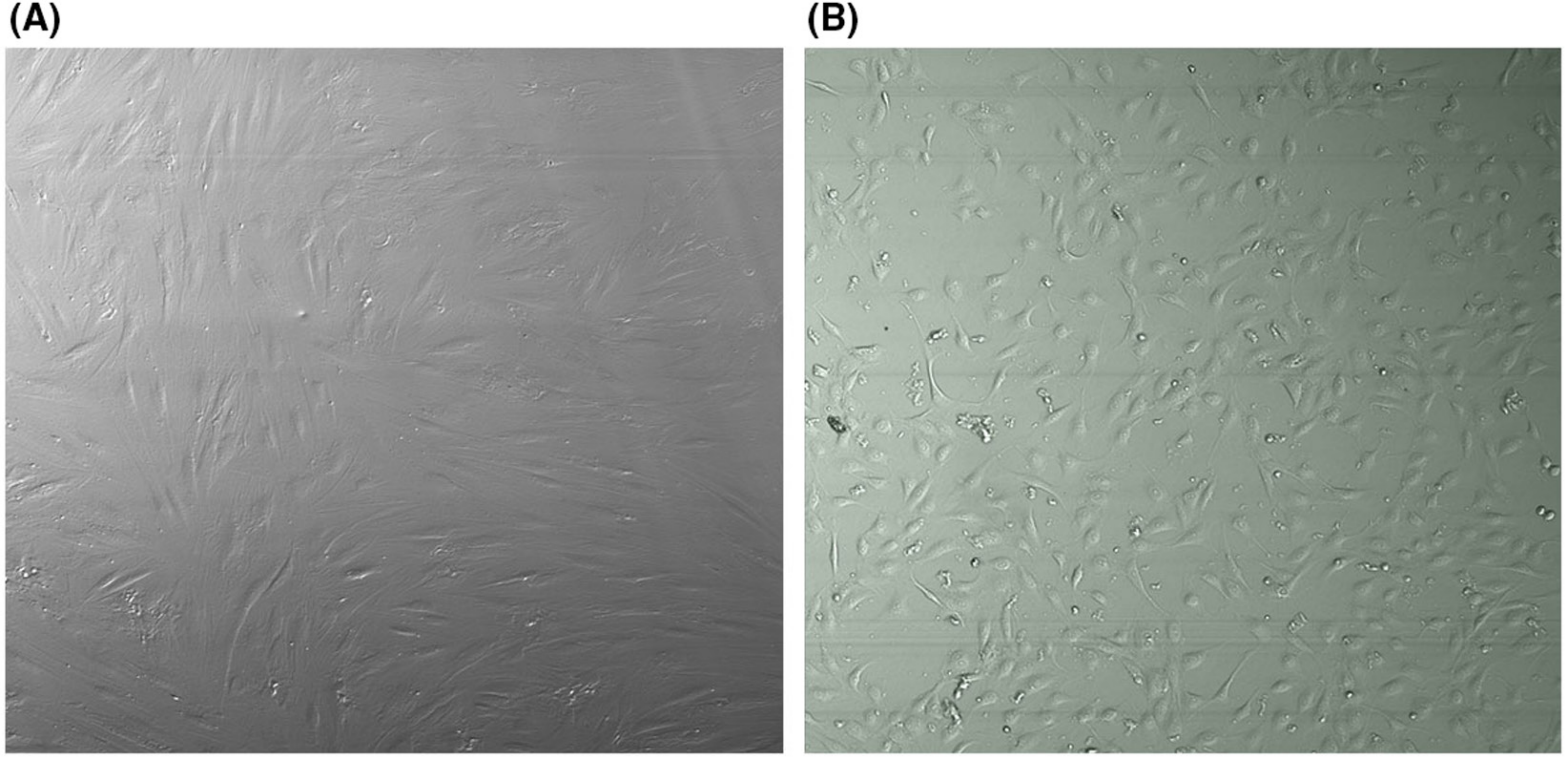
Characteristics of DMSCs and HUVECs. (A) Fibroblast‐like cell morphology of DMSCs. (B) Cobblestone‐like cell morphology of HUVECs

DMSCs were incubated in DMEM/F12 supplied with 10% FBS and 1% antibiotic at 37°C in a humidified atmosphere containing 5% CO_2_. The primary DMSCs attached to the bottom of the plates after 24–72 h incubation. At day 14–17, DMSCs showed typical fibroblast morphology like a spindle with multi‐layered flat cell bodies (Figure 2A). The surface markers of DMSCs derived from both psoriasis and healthy individual were positive for CD105, CD29, CD44, CD73 and CD90 and negative for CD45, CD34 and CD14 (Figure S2A). In this study, DMSCs were differentiated into adipocytes, osteoblasts and chondrocytes (Figure S2B–D) in DME/F12 medium with 10% FBS and additional supplementation of respective differentiation medium according to our previous experiments.^17^

HUVECs were incubated in EBM supplied with EGM‐2 at 37°C in a humidified atmosphere containing 5% CO_2_. The primary HUVECs attached to the bottom of the plates after 24 h incubation and the blood cells suspended in the medium. Then blood cells were removed through replaced fresh medium. After 3 to 5 days incubation, HUVECs showed cobblestone‐like morphology (Figure 2B). To further identify HUVECs, the expression of surface marker CD31 was tested by flow cytometry. Consistence with HUVECs identification, the surface marker of the isolated cells was positive CD31 (Figure S2E).

### 3.3 DMSCs‐derived EDIL3 stimulated the expression of αvβ3 in HUVECs

**FIGURE 3:**
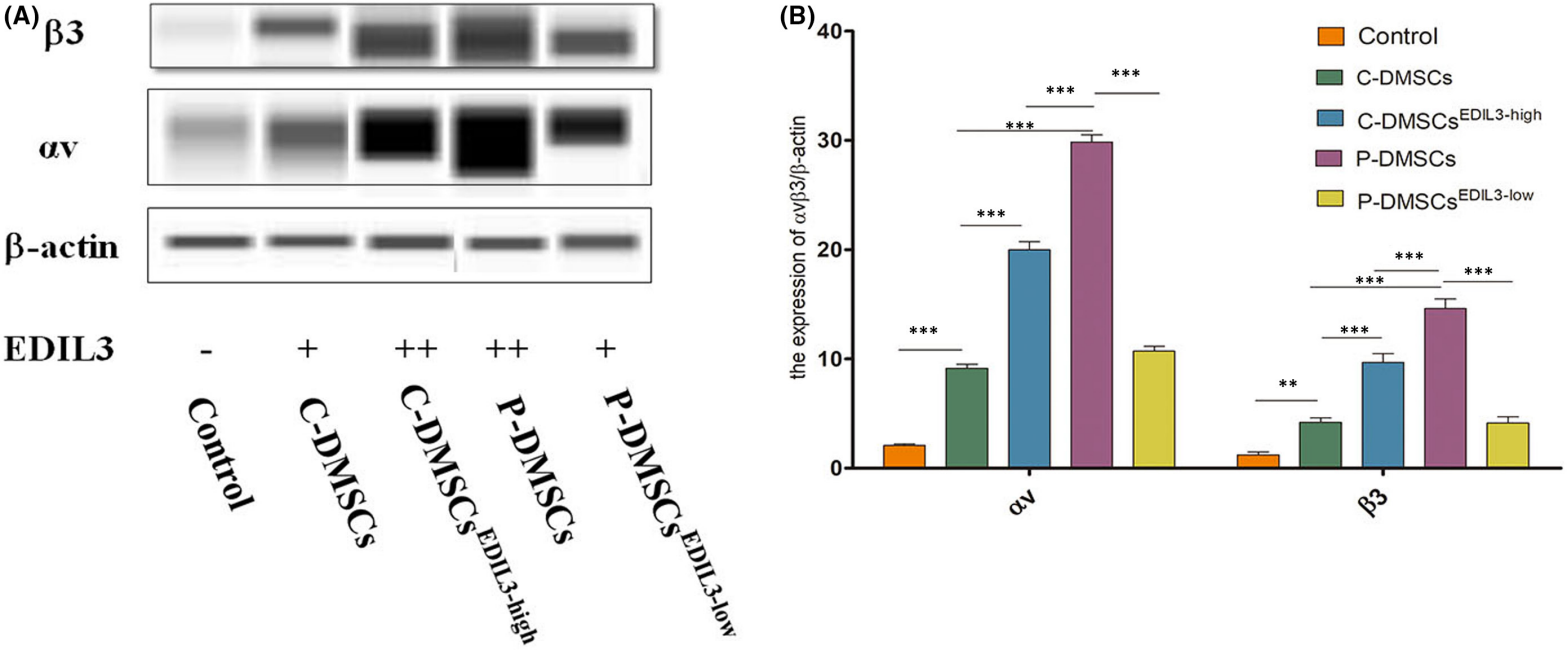
DMSCs‐derived EDIL3 led to increase the expression of αvβ3 in HUVECs. (A) The molecular weights of integrins αv, β3 and β‐actin were, respectively, 165 kDa, 129 kDa and 48 kDa. (B) Quantified results of αvβ3. *p* < 0.05 considered to be statistically significant (*p < 0.05, **p < 0.01, ***p < 0.001)

To determine the optimized time range of lower expression of EDIL3 induced by si‐RNA, at 48 h, 72 h and 96 h, the respective P‐DMSCs transfected were collected for western blot. Our data showed that the protein expression of EDIL3 was lower at 48 h; however, it recovered at 96 h (Figure S3). Thus, the subsequent co‐cultured incubation was all carried out for 48 h. And, C‐DMSCs were transfected using recombinant EDIL3 lentiviral vector, most C‐DMSCs positively expressed GFP (Figure S4) that showed successful lentiviral vectors transfection of C‐DMSCs. After co‐culture, we detected the expression of αvβ3 using western blot. The images revealed that both P‐DMSCs and EDIL3 overexpressed C‐DMSCs upregulated the expression of αvβ3 (*p* < 0.05) (Figure 3A,B). These data confirmed that DMSCs‐derived EDIL3 effectively promoted the expression of integrin αvβ3. Moreover, knockdown of EDIL3 in P‐DMSCs obviously reduced the expression of αvβ3 (*p* < 0.05). We also found compared with the controls, the expression of αvβ3 was upregulated in all co‐cultured groups (*p* < 0.05) (Figure 3A,B). And in P‐DMSCs group, the expression of αvβ3 was higher than C‐DMSCs^EDIL3‐high^ (*p* < 0.05) (Figure 3A,B). Together, these studies confirmed that not only C‐DMSCs^EDIL3‐high^ upregulated integrin αvβ3 but also the increase of αvβ3 in P‐DMSCs was more evident.

### 3.4 DMSCs‐derived EDIL3 activated the FAK/MEK/ERK signal pathway in HUVECs

**FIGURE 4:**
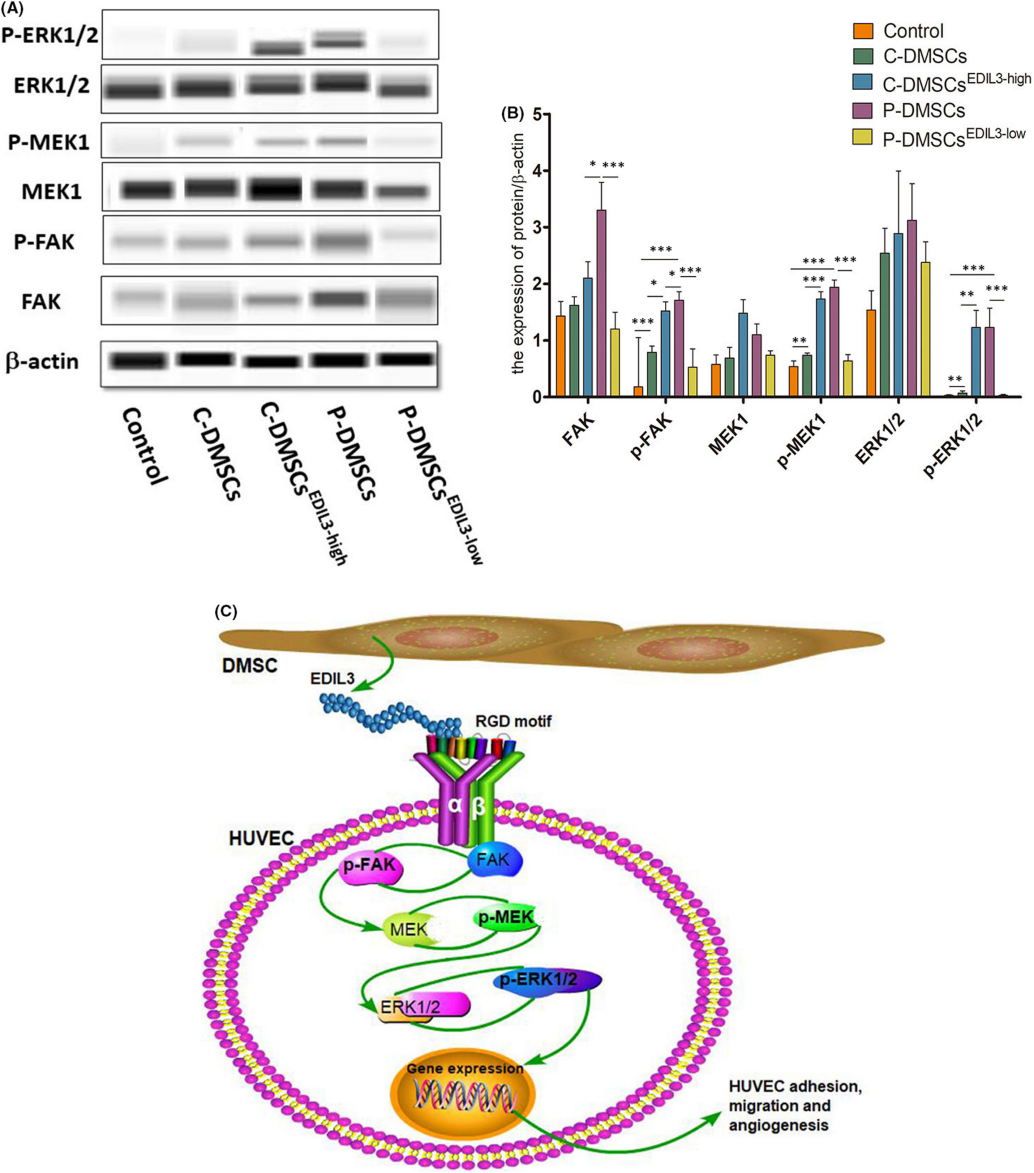
DMSCs‐derived EDIL3 activated FAK/MEK/ERK signal pathway in HUVECs. (A) The images of protein bands of β‐actin, FAK/p‐FAK, MEK/p‐MEK, ERK1/2 and p‐ERK1/2. The molecular weights were, respectively, 49 kDa, 116 kDa, 61 kDa, 50 kDa, 45 kDa, 44 kDa and 45 kDa. (B) Quantitative analysis of the protein levels. (C) The molecule model of regulating ECs functions by DMSCs‐derived EDIL3. P‐DMSCs secreted EDIL3, which upregulated integrin αvβ3 expression through RGD‐motif structure in HUVECs. Then, the downstream FAK was activated by integrins and translated into phosphorylated FAK (p‐FAK). The p‐FAK further activated MEK and ERK1/2. The integrin pathway transduced the signal into cell nucleus and regulated genes expression

Extracellular matrix proteins can activate intracellular signal cascades for various cellular events through activation of their receptors. Interaction of integrin with extracellular matrix proteins generates important intracellular signals for growth, survival and migration.^18^ We found that FAK/MEK/ERK signalling pathway can regulate endothelial proliferation, adhesion and migration in KEGG (https://www.kegg.jp/). To verify whether DMSCs‐derived‐EDIL3 induced changes of FAK, MEK and ERK depended on activation of integrin αvβ3, after co‐culture, we analysed the protein expressions of FAK/p‐FAK, MEK/p‐MEK, ERK1/2 and p‐ERK1/2 in HUVECs. We found that the total protein of MEK, ERK in different group was no significantly different (Figure 4A). Co‐incubation of P‐DMSCs or overexpressed EDIL3 C‐DMSCs with HUVECs for 48 h induced a significant increase of p‐FAK, p‐MEK and p‐ERK1/2 proteins in HUVECs (Figure 4B). And EDIL3 knockdown P‐DMSCs decreased the expression of p‐FAK, p‐MEK and p‐ERK1/2 in HUVECs (Figure 4A,B). In addition, in overexpressed EDIL3 group and P‐DMSCs group, we found that p‐FAK, p‐MEK and p‐ERK1/2 were upregulated in HUVECs (Figure 4A). Both the P‐DMSCs and C‐DMSCs^EDIL3‐high^ groups stimulated the expression of p‐MEK and p‐ERK1/2, but no significant changes of the both groups (Figure 4B). Then, the p‐FAK in P‐DMSCs group was significantly increased than C‐DMSCs^EDIL3‐high^ (Figure 4B). After knockdown of EDIL3 in P‐DMSCs, we found the p‐FAK, p‐MEK and p‐ERK1/2 proteins were all significantly decreased (Figure 4B). All data suggested the activation of FAK/MEK/ERK signal pathway in HUVECs, which was positively regulated through EDIL3 and αvβ3 (Figure 4C).

### 3.5 DMSCs‐derived EDIL3 through the αvβ3‐FAK/MEK/ERK axis induced tube formation of HUVECs

**FIGURE 5:**
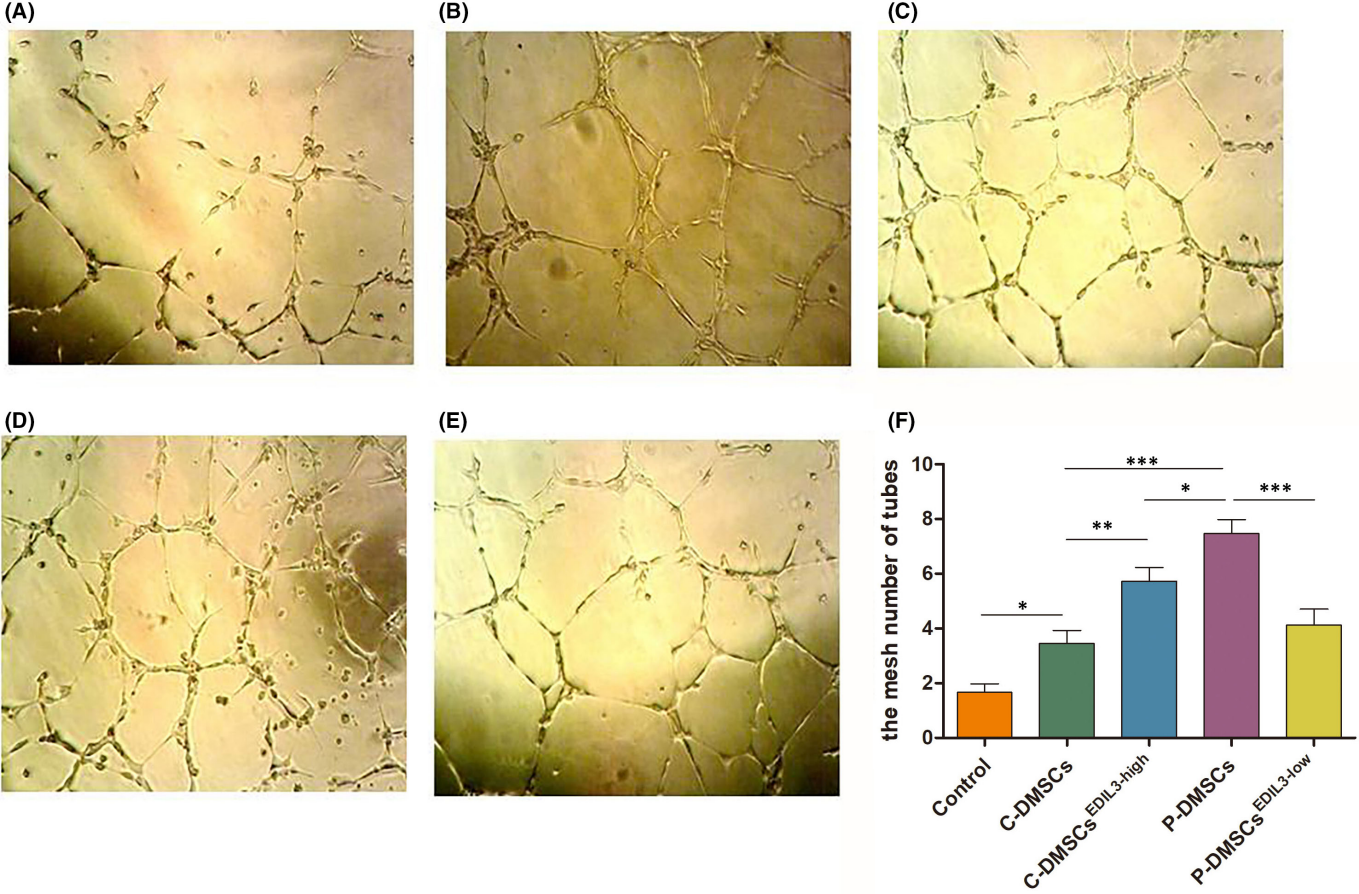
DMSCs‐derived EDIL3 promoted HUVECs tube formation in vitro. (A–E) After co‐culture, HUVECs were seeded onto Matrigel in 96‐well plates for 6 h and the tubes gradually formed in the (A) control, (B) C‐DMSCs, (C) C‐DMSCs^EDIL3‐high^, (D) P‐DMSCs and (E) P‐DMSCs^EDIL3‐low^ groups. Pictures were taken and the numbers of the tube meshes were measured. (F) Quantitative analysis of the number of tubes meshes

The process of tube formation is a result of dynamic reorganization of vascular system in vitro and is the characteristic trait of ECs. HUVECs belong to ECs and have the ability of tube formation in vitro. This study further confirmed that our protocols isolating HUVECs was successful and feasible (Figure 5A). The number and stability of tube mesh formation was found to be increased in co‐culture groups. After 48 h, the control no treatment with DMSCs displayed very few branches; however, the prominent branching networks were observed in DMSCs‐treated groups (Figure 5A–E) and the increase was significant (all *p* < 0.05) (Figure 5F). Tube‐forming ability in C‐DMSCs, as measured by the number of meshes assembled, was significantly decreased compared to that in P‐DMSCs (p < 0.001) (Figure 5F). In EDIL3 overexpressed group, after co‐culture, sprouting HUVECs were numerous and tubes were longer compared to these in C‐DMSCs group (Figure 5 B‐F) (p < 0.01). Knockdown of EDIL3 in P‐DMSCs significantly inhibited the tube formation of HUVECs compared to that in P‐DMSCs (*p* < 0.001) (Figure 5F). A similar effect was observed in EDIL3 overexpressed C‐DMSCs and P‐DMSCs; however, the increased tube meshes in P‐DMSCs were more significant (*p* < 0.05) (Figure 5F). Combining all results, these indicated that DMSCs‐derived EDIL3 through the αvβ3‐FAK/MEK/ERK axis induced tube formation of HUVECs.

## 4 DISCUSSION

Psoriasis is not only inflammatory‐dependent but also angiogenesis‐dependent disease.^5^, ^19^ Cytokines produced by immune cells, keratinocytes and ECs perpetuate the inflammatory process via positive feedback loops. Whether it is the inflammatory cell or the angiogenesis that initiates psoriasis is not clear, which like a long‐standing ‘chicken or egg’ question. Microvascular changes in lesions of psoriasis include vascular enlargement, pronounced tortuosity and elongation, increased permeability and ECs proliferation in the superficial dermis.^20^ The expanded and tortuous microvascular in lesions is required for circulation of nutrients, signalling molecules, gas exchange, waste removal and to provide an enlarged endothelial surface area for inflammatory cell trafficking.^19^ The initiation or maintenance of the chronic inflammatory state in psoriasis may depend partly on mediators of the angiogenic pathway.^21^ It is generally admitted that vascular expansion and angiogenesis in psoriasis is vessel enlargement, elongation, increased tortuosity and sprouting angiogenesis from the pre‐existing vascular bed.^4^ However, both angiogenesis and microvascular tortuosity all originate from active of ECs. So, altered ECs functions are involved in pathological processes of psoriasis and play important role in disease perpetuation and maintenance.

Mesenchymal stem cells (MSCs) can differentiate into adipocytes, osteoblasts and chondrocytes, are self‐renewing and expandable stem cells.^22^ We isolated DMSCs from skins of psoriatic patients and healthy volunteers and the results showed its surface markers were same as MSCs. And DMSCs also differentiated into adipocytes, osteoblasts and chondrocytes. CD31 is a specific marker of ECs^23^ and ECs can form capillary‐like structures plated on the Matrigel matrix in vitro, which can be used to define ECs.^24^ In present study, we successfully isolated DMSCs and HUVECs and co‐cultured them to evaluate the effect of DMSCs‐derived EDIL3 on ECs.

Our previous study demonstrated that psoriatic DMSCs promoted cell–cell adhesion and migration of ECs, but the molecular mechanism has not been explored.^12^ Our microarray analysis (data deposited under NCBI GEO GSE42632) showed that DMSCs from psoriatic skin lesions displayed significantly high expression of EDIL3.^17^ Previous studies have reported that EDIL3 is a recently cloned and characterized unique matrix protein that is expressed during early embryogenesis in ECs,^25^ and its expression is downregulated in later developmental stages. In healthy adult tissue, EDIL3 becomes quiescent or may no longer be expressed.^17^, ^26^, ^27^ However, EDIL3 expression can be re‐initiated during ischemia and is upregulated in tumour vascular tissues.^25^, ^26^ These findings suggested that EDIL3 may have an important role in mediating aberrant vascular remodelling. We further analysed the mRNA and protein of EDIL3 in P‐DMSCs using RT‐qPCR and western blot. The results showed mRNA and protein expression of EDIL3 in psoriatic DMSCs was markedly higher than healthy.^9^ To further explore the role of EDIL3 in psoriasis, in present study, we detected the epidermis thickness and microvessel density in IMQ‐induced mouse model through injecting EDIL3. Our studies found that the epidermis thickness and microvessel density were both elevated in the IMQ‐induced psoriasis‐like mouse models. The increased microvascular in lesions is required for epidermis cells to circulate nutrients, molecules, gas exchange and waste removal.^27^ The hyperplasia of the epidermis and maintenance depend on abnormal elongation and angiogenesis of microvascular. The results suggested that EDIL3 accelerated the process of psoriasis through induce angiogenesis of microvascular.

Based on previous studies, we found the DMSCs and EDIL3 play an important role in psoriasis. To clarify the mechanism of the effect of DMSCs‐derived EDIL3 on ECs, we explored the downstream pathway associated with EDIL3. We found that EDIL3 increased expression of integrin αvβ3 in HUVECs. Through KEGG (https://www.kegg.jp/), we found ERK signal pathways have been implicated in cell migration and adhesion. After co‐culture with the silenced or overexpressed EDIL3 DMSCs, we detected the protein of FAK/MEK/ERK signal pathway in HUVECs. Our results demonstrated that after stimulated by DMSCs‐derived EDIL3, except for the αvβ3 receptor, FAK/MEK/ERK signal pathway was activated.

Sprouting angiogenesis begins with the dissolution of the basement membrane, followed by the migration and attachment of endothelial cells (ECs) and consequently forms a loop structure. After co‐culture, we analysed the numbers of the tube meshes of HUVECs in vitro. The numbers of meshes were significantly increased in C‐DMSCs^EDIL3‐high^ and psoriasis groups. Our data indicated that DMSCs‐derived EDIL3 contributed to activating ECs properties, then promoted tube‐structure formation and caused angiogenesis in dermal of psoriasis. Compared with the control, the tube meshes of HUVECs in P‐DMSCs^EDIL3‐low^ group was decreased but without significantly different. The studies of angiogenesis have shown that the process occurs via cell motility, migration and division to form sprouts, and the resulting hollow vascular tubes anastomose to form capillary loops.^28^ Cells motility and migration play important roles during angiogenesis. The result may mainly be caused by decreased migration of ECs.

Analysis of HUVECs following DMSCs‐derived EDIL3 treatment revealed increased αvβ3‐linked kinases such as increased p‐FAK, p‐MEK and p‐ERK, consistent with increased cell tube formation. Taken together, these data suggested that DMSCs‐derived EDIL3 mediated ECs tube formation through RGD‐motif interaction with the integrin αvβ3 receptors. The αvβ3 could interact with EDIL3, activating FAK/MEK/ERK signal pathway, transferring the extracellular signalling into cytosol and affecting the expression of nuclei DNA and cytoskeleton. Extracellular matrix proteins can activate intracellular signal cascades for various cellular events through activation of their receptors. Interaction of integrin with extracellular matrix proteins generates important intracellular signals for growth, survival and migration.^18^ Intracellular signals from integrins induce the formation of a focal adhesion complex, which is critical for cell adhesion and migration.^29^, ^30^ FAK is a tyrosine kinase plays a central role during the series of processes.^31^ FAK converts inactive Ras‐GDP to active Ras‐GTP, and the latter acts to mitogen activated protein kinase (MAPK or MEK) and extracellular signal‐regulated kinase‐1/2 (ERK1/2).^32^ FAK/MEK/ERK signal pathway is involved in many cellular functions including cell survival and migration and inhibition of this pathway has been shown to halt the migration of a variety of cell types.^33^ In addition, ERK promotes the transcription of a variety of genes, many of which involved in motility.^33^ In the present study, we demonstrated that DMSC‐derived‐EDIL3 involved in EC angiogenesis and the function acted via the αvβ3‐ FAK/MEK/ERK signal pathway.

In conclusion, EDIL3 was upregulated in P‐DMSCs and was an important pro‐angiogenic protein. In present study, we found EDIL3 accelerated the process of psoriasis in vivo. We also demonstrated that DMSCs‐derived EDIL3 induced angiogenesis of HUVECs in vitro through αvβ3‐FAK/MEK/ERK signal pathway. The overexpressed EDIL3 in P‐DMSCs induced the activation of the signalling pathway can be inhibited by si‐RNA, in accordance with the inhibiting the capabilities of angiogenesis of ECs. This suggested that EDIL3 from DMSCs and αvβ3‐FAK/MEK/ERK signal pathway in ECs play an important role in the pathogenesis of psoriasis. The essential step in occurrence and development of psoriasis, based on this, we speculated that EDIL3 and αvβ3‐FAK/MEK/ERK signal pathway will provide a valuable therapeutic target to control angiogenesis.

## AUTHOR CONTRIBUTIONS

**Xuping Niu:** Formal analysis (equal); project administration (equal); writing – original draft (equal). **Qixin Han:** Data curation (equal); formal analysis (equal); writing – original draft (equal); writing – review and editing (equal). **Xinhua Li:** Methodology (equal); software (equal). **Juan Li:** Methodology (equal). **Yanmin Liu:** Methodology (equal). **Yan Li:** Writing – review and editing (equal). **Yan Wu:** Funding acquisition (equal); writing – original draft (equal); writing – review and editing (equal). **Kaiming Zhang:** Project administration (equal).

## FUNDING INFORMATION

This work was supported by grants from National Natural Science Foundation of People Republic of China (No. 81803146).

## CONFLICT OF INTEREST

All authors indicated no potential conflicts of interest.

## Supporting information


Figure S1

Figure S2

Figure S3

Figure S4

Table S1

Table S2

## Data Availability

The data sets used and/or analysed during the current study are available from the corresponding author on reasonable request.
