# Supplementary figures and images for: EDIL3 influenced the αvβ3‐FAK/MEK/ERK axis of endothelial cells in psoriasis
Source: J Cell Mol Med. 2022 Sep 6;26(20):5202–12. doi: 10.1111/jcmm.17544 (PMC9575107; doi:10.1111/jcmm.17544)

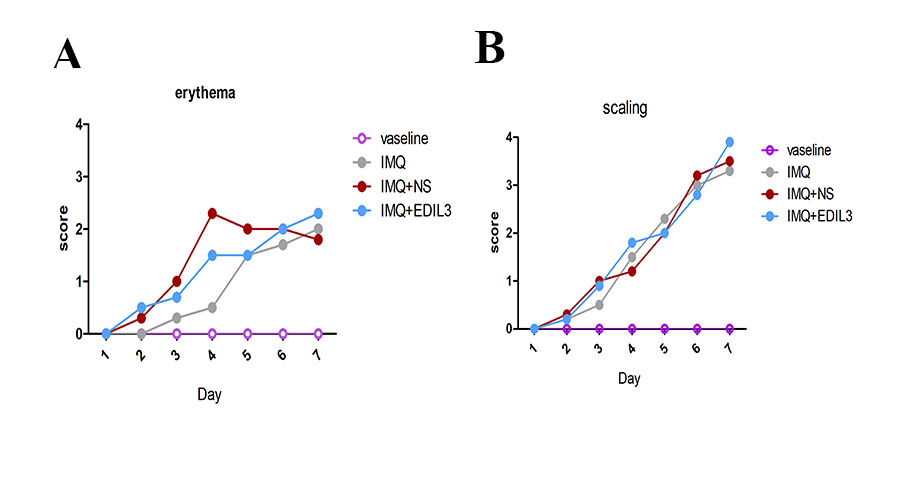

Supplement: Supplementary file 1 — Figure S1 [file JCMM-26-5202-s005.jpg]

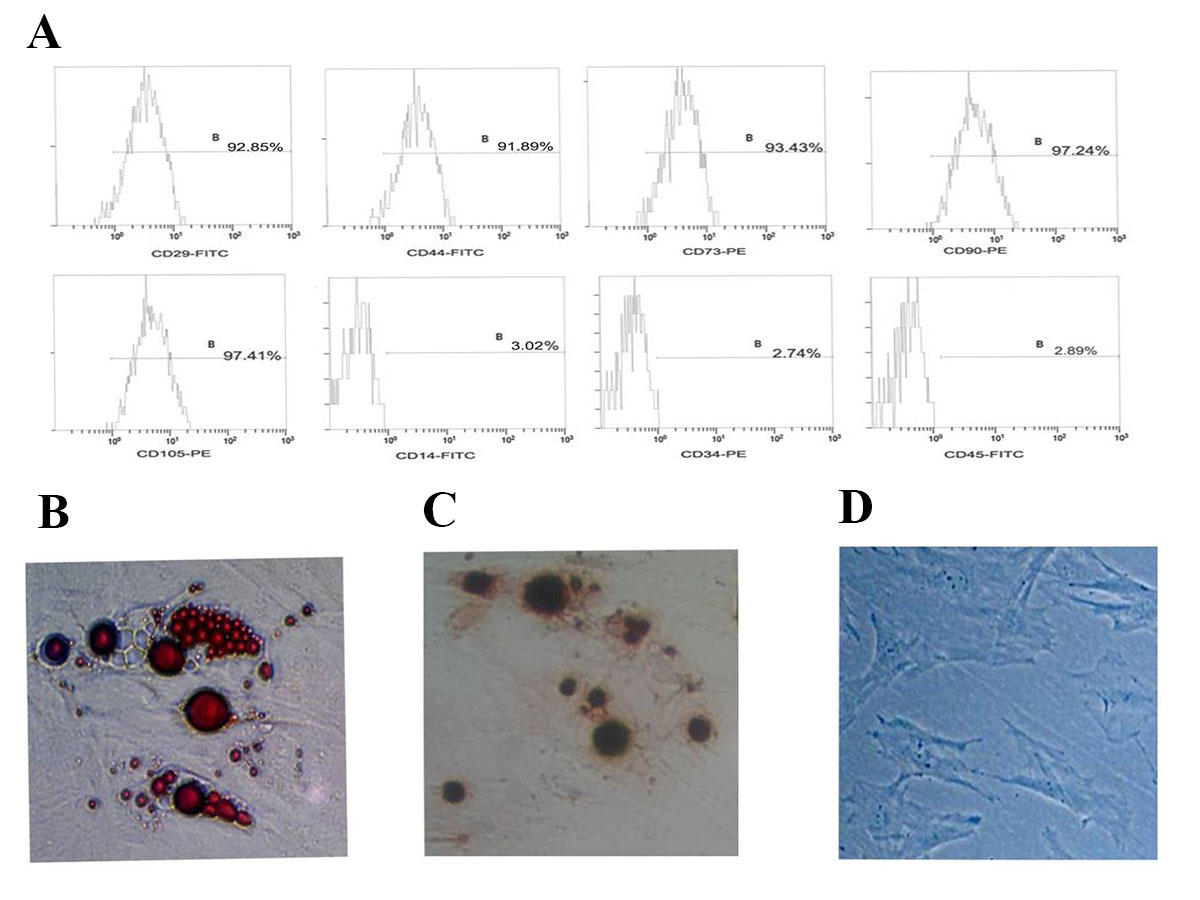

Supplement: Supplementary file 2 — Figure S2 [file JCMM-26-5202-s002.zip › JCMM_17544_Suppl. Fig.2A-D.jpg]

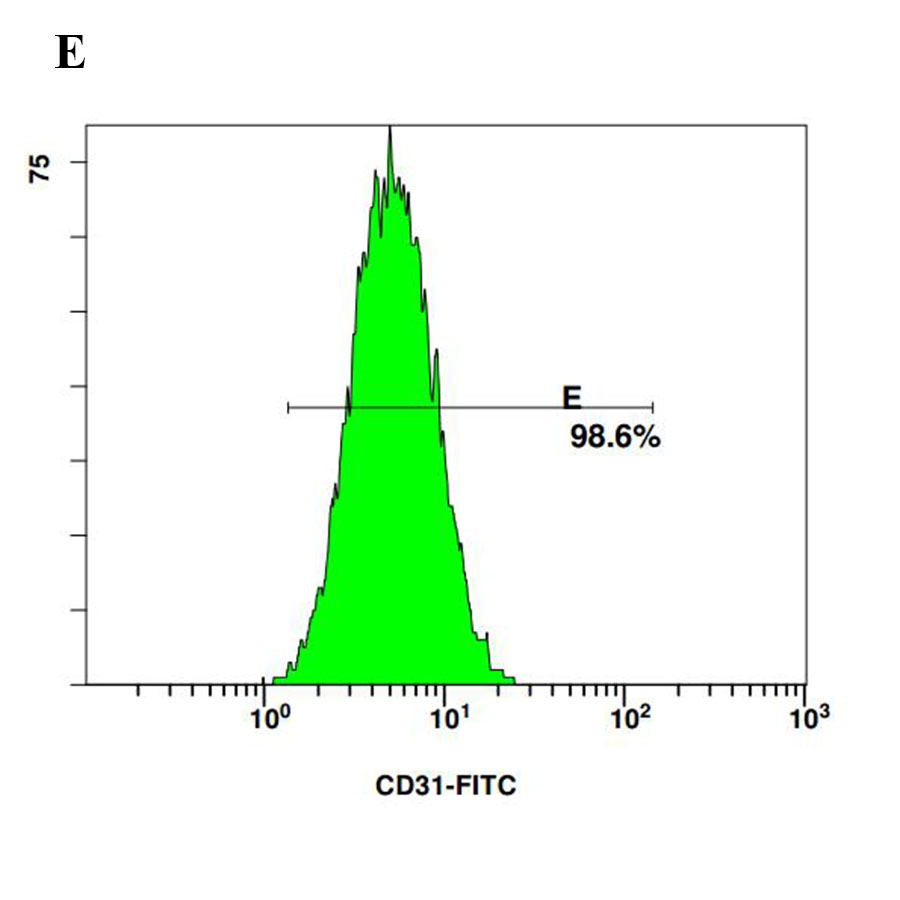

Supplement: Supplementary file 2 — Figure S2 [file JCMM-26-5202-s002.zip › JCMM_17544_Suppl. Fig.2E.jpg]

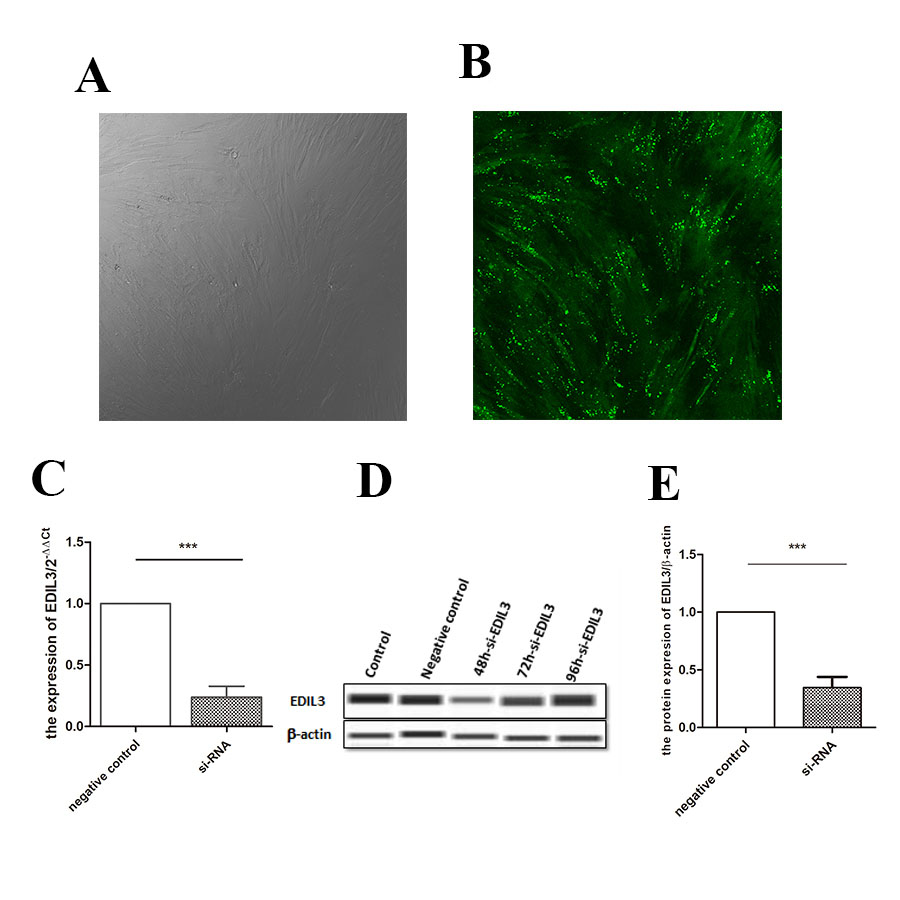

Supplement: Supplementary file 3 — Figure S3 [file JCMM-26-5202-s001.jpg]

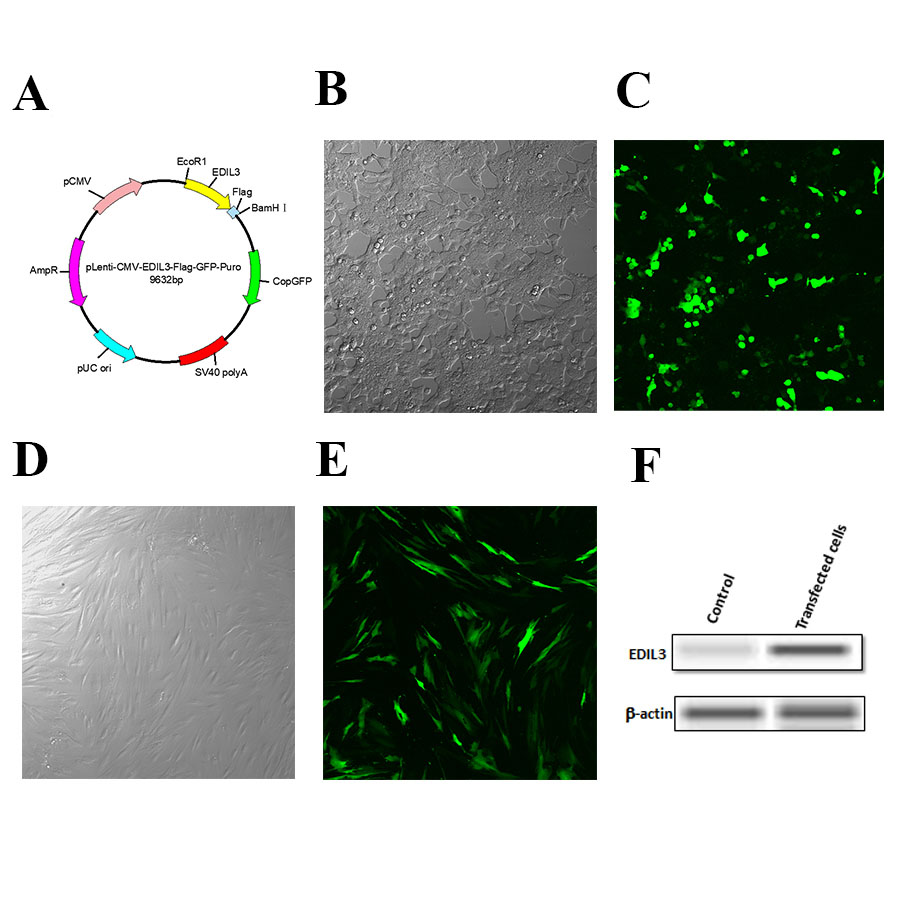

Supplement: Supplementary file 4 — Figure S4 [file JCMM-26-5202-s004.jpg]

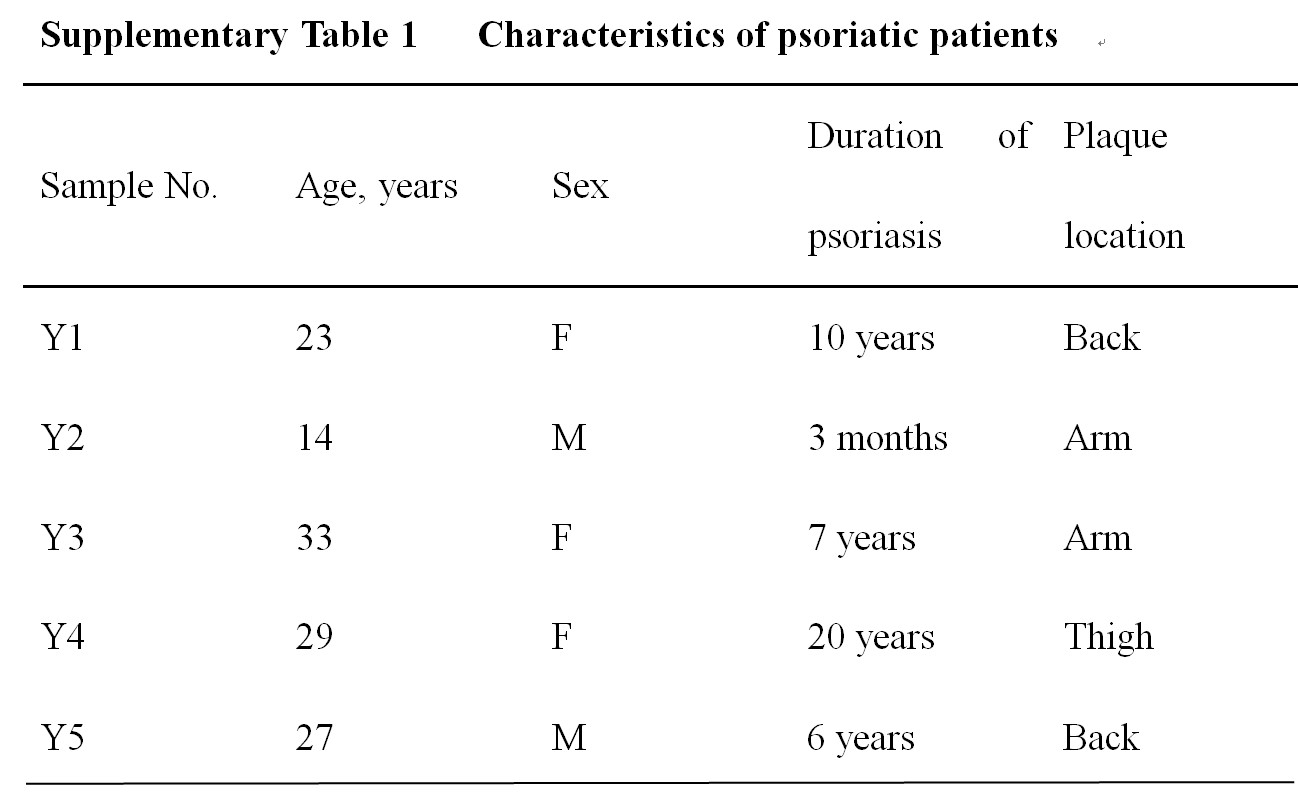

Supplement: Supplementary file 5 — Table S1 [file JCMM-26-5202-s003.jpg]

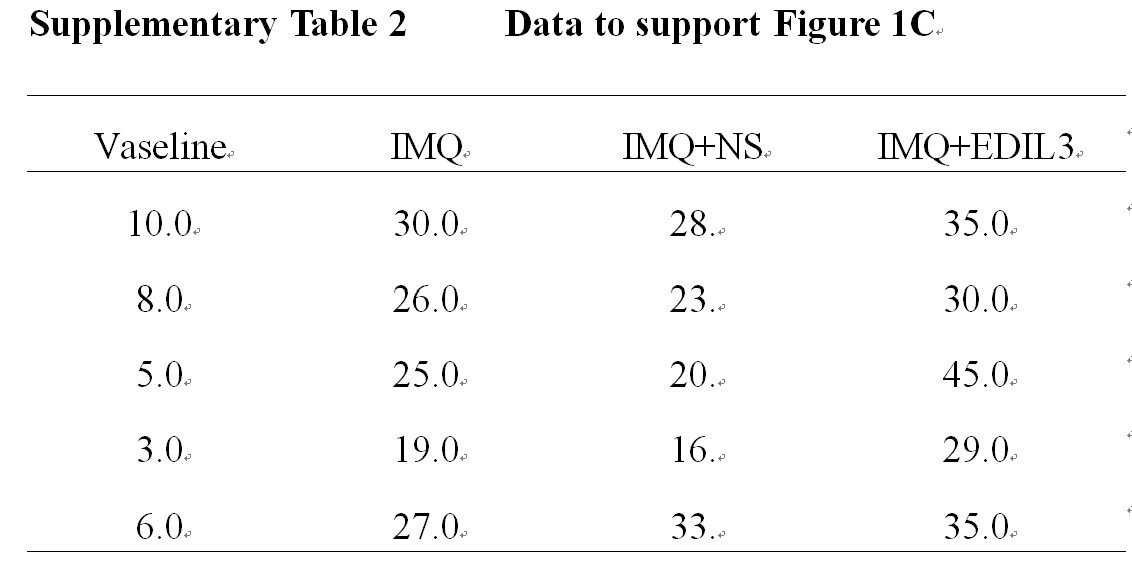

Supplement: Supplementary file 6 — Table S2 [file JCMM-26-5202-s006.jpg]
